# Selected Nutrients and Their Implications for Health and Disease across the Lifespan: A Roadmap

**DOI:** 10.3390/nu6126076

**Published:** 2014-12-22

**Authors:** Szabolcs Péter, Manfred Eggersdorfer, Dieneke van Asselt, Erik Buskens, Patrick Detzel, Karen Freijer, Berthold Koletzko, Klaus Kraemer, Folkert Kuipers, Lynnette Neufeld, Rima Obeid, Simon Wieser, Armin Zittermann, Peter Weber

**Affiliations:** 1 DSM Nutritional Products Ltd., Wurmisweg 576, 4303 Kaiseraugst, Switzerland; E-Mails: manfred.eggersdorfer@dsm.com (M.E.); peter.weber@dsm.com (P.W.); 2 University Medical Center Groningen, University of Groningen, Hanzeplein 1, 9700 RB Groningen, The Netherlands; E-Mails: e.buskens@umcg.nl (E.B.); f.kuipers@umcg.nl (F.K.); 3 Department of Geriatric Medicine, Medical Center Leeuwarden, Henri Dunantweg 2, 8934 AD Leeuwarden, The Netherlands; E-Mail: Dieneke.van.Asselt@ZNB.NL; 4 Nestlé Research Centre, Vers-chez-les Blanc, 1000 Lausanne, Switzerland; E-Mail: patrick.detzel@rdls.nestle.com; 5 Medical Department, Nutricia Advanced Medical Nutrition, Amsterdam, The Netherlands; E-Mail: karen.freyer@nutricia.com; 6 Department of Public Health and Primary Care (CAPHRI), University of Maastricht, Universiteitssingel 40, 6229 ER Maastricht, The Netherlands; 7 Dr. von Hauner Children’s Hospital, Ludwig-Maximilians-University of Munich, Lindwurmstr. 4, 80337 Munich, Germany; E-Mail: Berthold.Koletzko@med.uni-muenchen.de; 8 Sight and Life, Wurmisweg 576, 4303 Kaiseraugst, Switzerland; E-Mail: klaus.kraemer@dsm.com; 9 Department of International Health, Johns Hopkins Bloomberg School of Public Health, 615 N Wolfe St #5041, Baltimore, MD 21205, USA; 10 Micronutrient Initiative, 180 Elgin Street, Suite 1000, K2P 2K3 Ottawa, Ontario, Canada; E-Mail: lneufeld@gainhealth.org; 11 Aarhus Institute of Advanced Studies, University of Aarhus, Aarhus, Dk-8000, Denmark; E-Mail: rimaobeid@aias.au.dk; 12 Winterthur Institute of Health Economics, Gertrudstrasse 15, 8401 Winterthur, Switzerland; E-Mail: simon.wieser@zhaw.ch; 13 University of Bochum, Georgstraße 11, 32545 Bad Oeynhausen, Germany; E-Mail: azittermann@hdz-nrw.de; 14 University of Bonn, Endenicher Allee 11-13, 53115 Bonn, Germany; 15 University Hohenheim, Schloß Hohenheim 1, 70599 Stuttgart, Germany

**Keywords:** healthy ageing, global health, nutrition economics, undernutrition

## Abstract

Worldwide approximately two billion people have a diet insufficient in micronutrients. Even in the developed world, an increasing number of people consume nutrient-poor food on a regular basis. Recent surveys in Western countries consistently indicate inadequate intake of nutrients such as vitamins and minerals, compared to recommendations. The International Osteoporosis Foundation’s (IOF) latest figures show that globally about 88% of the population does not have an optimal vitamin D status. The Lancet’s “Global Burden of Disease Study 2010” demonstrates a continued growth in life expectancy for populations around the world; however, the last decade of life is often disabled by the burden of partly preventable health issues. Compelling evidence suggests that improving nutrition protects health, prevents disability, boosts economic productivity and saves lives. Investments to improve nutrition make a positive contribution to long-term national and global health, economic productivity and stability, and societal resilience.

## 1. Introduction

According to the World Health Organization (WHO), in the majority of countries, the proportion of people aged over 60 years is growing faster than any other age group, as a result of longer life expectancy and declining fertility rates. Consequently, from 2000 until 2050, the proportion of the global population over 60 years will double from ca. 11% to 22% [1]. This can be interpreted as a success story for public health policies and for socioeconomic development, but at the same time it also challenges societies to adapt and overcome the health and economic burden of ageing and age-related conditions. Healthy ageing is key if older people are to remain independent and to play an integral part in society. (“Healthy” refers to physical, mental, and social wellbeing as indicated in the WHO definition of health.) Life-long health promotion, with a clear emphasis on the fundamental role of nutrition and micronutrients to reduce risks of chronic conditions and disability, may prevent or delay the onset of several non-communicable diseases. One example for such an initiative in the European Union is the European Innovation Partnership on Active and Healthy Ageing, which aims to increase the average healthy lifespan by two years by 2020 [2].

In the long-term, inadequate micronutrient intake can lead to chronic micronutrient undernutrition and consequent health problems, including lower resistance to development of chronic disease thus reducing health span and longevity. Micronutrient deficiencies (MNDs) are well established in the developing world, however, a growing body of evidence shows the existence of MNDs also in the developed world [3]. The term ‘hidden hunger’ is commonly used in this context, as these deficiencies are often ignored and underestimated despite the serious consequences they can hold for individuals, communities, and national economies. MNDs are particularly harmful during pregnancy and early childhood due to their impact on the physical and cognitive development of children. Micronutrient requirements also have to be adjusted later in life, for example to the specific micronutrient requirements of the elderly and those living in particular settings such as hospitals and institutions.

Scientists, clinicians, and public health specialists met at the European Research Institute for the Biology of Ageing (University Medical Center Groningen, The Netherlands), in a workshop to address topics related to the contribution of nutrients to healthy ageing, the impact of nutritional approaches in health and disease and solutions on healthy ageing, and the implications of optimal nutrient intake over the life course on healthcare-related costs including possible economic benefits. The workshop covered a wide range of topics, from health economics of disease-related malnutrition through healthy ageing during the course of life to health and economic benefits of fortification or supplementation with certain nutrients. A roadmap has been proposed to address pending issues that must contribute to reduction of health care costs.

## 2. Health Economics of Malnutrition

There are several definitions of malnutrition. An international acknowledged definition is “A state of nutrition in which a deficiency, excess or imbalance of energy, protein, and other nutrients causes measurable adverse effects on tissue/body form (body shape, size and composition) function, and clinical outcome” [4]. Malnutrition thus includes both overnutrition (too many nutrients) and undernutrition (insufficient nutrition). In this manuscript, malnutrition refers to undernutrition. Furthermore, there are several causes for malnourishment. Depending on the cause, one can talk about disease-related malnutrition, wasting, sarcopenia or nutrient depletion like MND [5].

### 2.1. Measuring the Impact of Malnutrition

Measuring the effects of malnutrition is important to assess the burden of disease in populations, to analyze trends and progress over time, and to compare countries, regions and social groups. It is also vital as a priority-setting tool for policy makers. The global consensus recognizing the value of appropriate nutrient intake is based on a solid body of evidence and on a number of powerful nutritional and economic models. Examples of health economic evaluations are cost-utility analyses in which the values are expressed as Quality-Adjusted Life Years (QALYs) and Disability Adjusted Life Years (DALYs). These analyses attach utility weights, so that morbidity and mortality can be combined in a common metric. These are particularly useful for making comparisons across health care interventions and disease domains. QALYs combine quantity and quality of life in a single index: One year of life × 1 utility value = 1 QALY. The utility for perfect health = 1, the utility for death = 0. The utility weights underpinning the QALY are based in part on subjective assessment or individual preferences. This subjective assessment of quality of life is sometimes referred to as “utility”. Already in 2006, the National Institute for Health and Clinical Excellence (NICE) developed a model to estimate the incremental cost/QALY for nutritional screening and management of undernutrition by oral nutritional supplements compared to the situation where no such program would be in place for the elderly, resulting in £6800/QALY. As a threshold of £20–30,000/QALY is used by NICE for considering treatments to be cost-effective, screening and management of undernutrition can therefore be considered as value for money [6].

The DALY measures burden of disease, indicating how many years of healthy life are lost due to no-fatal illness or impairment or death. The DALY also reflects the number of individuals that are ill or die in each age-sex group and location in a certain population. Where YYL = years of life lost due to premature mortality in the population (number of deaths multiplied by standard life expectancy at age of death in years) and YLD = years lost due to disability (number of disability cases multiplied by the average duration of disability weighted by the severity of a particular disease), then DALY = YYL + YLD. DALYs are based on egalitarian principles: the same life expectancy and disability weights are used regardless of geography, gender, and race. DALYs form the basis of WHO’s Burden of Disease concept, as they offer a comprehensive and comparable assessment of mortality and loss of health due to diseases, injuries, and risk factors for all regions of the world. DALYs have provided a powerful tool to measure and track the contribution of nutritional deficiencies to health—which is critical to raise awareness. Using this for policy making and priority setting presents a number of important challenges.

DALYs and QALYs are examples for measuring effectiveness and subsequently cost-effectiveness of specific policies and/or interventions for policy making and priority setting, showing the efficiency across different age and sex groups. For example, they can be used in assessing the cost-effectiveness of mandatory food fortification with folic acid. In a recent study, mandatory fortification with folic acid was found to be cost-effective in Australia [7]. The cost-effectiveness evaluations of specific policies have been successful in solving the virtual elimination of goiter through salt iodization and the improvement of life expectancy of patients with metabolic disorders by ready-to-use therapeutic foods. However, there have also been limited successes in programs aiming at prevention of stunting, anemia, obesity, and associated co-morbidities. Failures have been partly due to the oversimplification of complex problems. Although needed, complex solutions may not rank high in current cost effectiveness analyses. There needs to be caution against generalization, and it is important to strengthen methods and models to fit the needs of complex nutritional problems. Early investments are necessary for long-term gains: this has been recognized in many social protection programs. Improvements in nutrition require strong efforts to change behavior by education and not just by providing a cost-effective product.

### 2.2. The Global Impact of Inadequate Micronutrient Intake

Inadequate micronutrient intake can have serious health consequences for individuals, but also has a wider impact on societies, economies, and healthcare and welfare systems. A recent publication demonstrates that the use of specific dietary supplements among those consumers that are at a high risk of experiencing a costly disease-related event can lead to healthcare cost savings. For example, the total cumulative direct healthcare costs related to osteoporosis-attributed bone fractures among all US women over the age of 55 diagnosed with osteoporosis is expected to be nearly $136 billion from 2013 to 2020. An average of $1.87 billion per year and a cumulative savings of $15 billion from 2013 to 2020 in avoidable hospital utilization costs is potentially realizable if all US women over the age of 55 diagnosed with osteoporosis were to use calcium and vitamin D dietary supplements at preventive daily intake levels [8].

**Figure 1:**
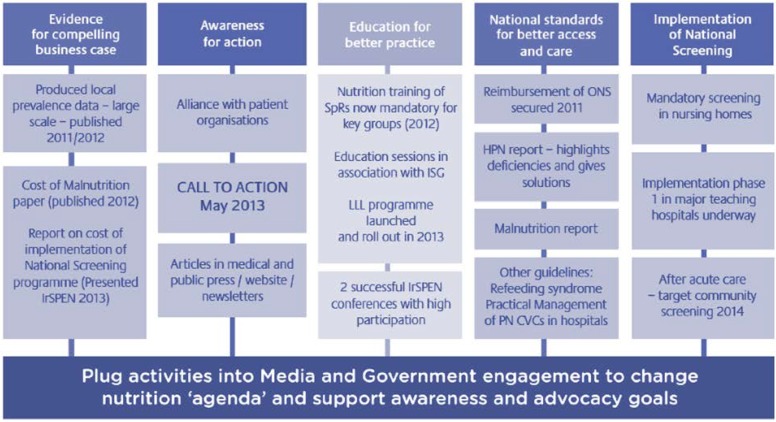
Irish Society for Clinical Nutrition and Metabolism (IrSPEN) adopted a highly focused strategy, drawing on ‘best practice’ examples, adapting and localizing key initiatives, and developing collaborations and partnerships to extend its sphere of influence.

It is widely recognized that MNDs are a significant public health problem. For example, globally one in two pre-school children suffers from anemia, many of them because of iron deficiency. Furthermore, about 190 million pre-school children suffer from vitamin A deficiency, but deficiencies in iodine, folate, and zinc are also highly prevalent. Wieser *et al.* carried out a burden of disease study, building a model of the lifetime consequences of micronutrient deficiencies and applying it to the Philippines. The study used data from two major surveys carried out in 2008: The Demographics and Health Survey (DHS) by the Philippines National Statistics Office (12,469 households) and the National Nutrition Survey (NNS) by the Philippines Food and Nutrition Research Institute (FNRI) (36,634 households). The population was classified into 10 socio–economic strata and two age groups *per strata* (children aged 6–23 and 24–59 months). Prevalence of MNDs was based on hemoglobin, serum retinol, and serum zinc levels assessed in the NNS. Different health consequences of deficiencies (iron, vitamin A, and zinc) were examined: mortality, morbidity, and limitation of physical and cognitive development. The study also looked at direct medical costs, short- and long-term production losses and intangible costs such as number of life years lost and quality of life lost. The time dimension was the lifetime costs of a 1-year cohort. The results showed that total lifetime costs amounted to medical costs of US $29 million, production losses of US $462 million and intangible costs of 116,656 DALYs. Costs are dominated by future lifetime costs due to impaired mental and physical development and costs of premature deaths over current losses. Costs are substantially higher in the 6–23 months age group. There are considerable differences between socioeconomic groups, with the potential revenues in the poorest decile of households being five times higher than in the wealthiest decile. Costs are much higher for vitamin A and iron deficiency than for zinc deficiency, with the exception of direct medical costs. Significantly, 83% of DALY losses and nearly all production losses due to iron deficiency occur in future lifetime. Without specific knowledge, therefore, parents may have no immediate reason to improve the diet of the child. The study concludes that policies aimed at reducing MNDs in children living in poor households should take account of the informational, behavioral, and economic obstacles. The high prevalence of MNDs in lower socio-economic strata may be one of the factors leading to a health-based poverty trap. Due to the impact of MNDs on physical and cognitive development, many children will not be able to reach their full potential and will become poor parents in their later life. The initiative “Fighting the malnutrition battle: the power of partnerships”, implemented by the Irish Society for Clinical Nutrition and Metabolism (IrSPEN) is a good example for the collaboration between academy, health care system, and industry partners. IrSPEN has managed to make rapid progress in its efforts to build a solid evidence base, increase malnutrition awareness, introduce nutrition training into post graduate medical education, and secure access to nutrition support for community patients (Figure 1) [9]. This approach can be adapted and used by other countries as well.

## 3. Healthy Ageing during Life Course

### 3.1. Translating Scientific Knowledge into Public Health Solutions

The European Commission’s European Innovation Partnership on Active and Healthy Ageing (http://ec.europa.eu/active-healthy-ageing) has been established to take the challenges of an ageing population and the associated challenges like lifestyle-related diseases, health workforce shortages, and the financial sustainability of health systems. The Partnership is part of the Innovation Union flagship initiative of the Europe 2020 strategy.

The healthcare policy of the European Commission is based on the Lisbon Treaty’s Article 168, which calls for ‘The attainment of a higher level of health protection through all European policies and activities’ and encourages cooperation between member states. The EU complements national policies towards improving public health, preventing physical and mental diseases, and promoting research, as well as information and education and monitoring and combating serious cross-border health threats. It also adopts a legislation to meet common safety concerns related to human components, the veterinary and phyto-sanitary fields and medical products and devices. Before discussing in more detail the European Innovation Partnership on Active and healthy Ageing, the EU health strategy in general, and the EU Platform for action on Diet, Physical activity, and health will be briefly discussed.

The EU Health Strategy “Together for health” (http://ec.europa.eu/health/strategy/policy/index_en.htm) supports the overall Europe 2020 strategy. It is an overarching policy framework designed to work towards good health in an ageing Europe, protecting citizens from health threats and supporting dynamic health systems and new technologies through legislation, cooperation, and financing. The Strategy for Europe on Nutrition, Overweight, and Obesity-related health issues (http://ec.europa.eu/health/nutrition_physical_activity/policy/strategy_en.htm) calls for action in six priority areas: 1. Better-informed consumers; 2. Making the healthy option available; 3. Encouraging physical activity; 4. Developing the evidence base to support policymaking; 5. Developing monitoring systems; 6. Putting children and low socio-economic groups as a priority.

The EU Platform for action on Diet, Physical Activity and Health (http://ec.europa.eu/health/nutrition_physical_activity/platform/index_en.htm) is a forum for European-level umbrella organizations, ranging from the food industry to consumer protection non-governmental organizations (NGOs). Its objective is to tackle current trends in diet and physical activity.

Europe 2020 is the EU’s inclusive growth strategy. To implement this strategy, Europe has identified the so-called flagship initiatives, including the Innovation Union flagship initiative. The European Innovation Partnership on Active and Healthy Ageing (EIP on AHA) is part of the Innovation Union flagship initiative and is a new method of collaboration that is focused on active and healthy ageing. The European Commission recognizes the challenge of the demographic change of an ageing society and the challenges this presents to society and economy. Total age-related spending (on education, pensions, healthcare, and unemployment benefits) is expected to increase by 4.75% points of Gross Domestic Product (GDP) by 2060. There is also a shrinking workforce in the care sector and an insufficient number of health professionals.

The goal of the European Innovation Partnership on Active and Healthy Ageing is to add on average two healthy life years by 2020. In a broader sense, the EIP on AHA pursues a triple win for Europe by improving the health and quality of life of European citizens with particular focus on older people, improving the long-term sustainability and efficiency of health and social care systems and enhancing the competitiveness of the EU-industry through business and expansion of new markets. It aims to achieve this through innovative collaboration through crosscutting, connecting, and engaging stakeholders across sectors, from the private and public sectors. It has established Action Groups (active partners working together towards a specific goal). Furthermore, there are Reference Sites, which provide the EIP on AHA with already implemented examples of a comprehensive, innovation-based approach to active and healthy ageing. These sites can be coalitions of regions, cities, integrated hospitals or care organizations that are able to show their impact and show particular innovative practices which could be transferred to other European contexts. Also a marketplace for innovative ideas (online collaboration, open to everybody) has been launched. One of the Action Groups is on the Prevention of Functional Decline and Frailty (http://ec.europa.eu/research/innovation-union/index_en.cfm?section=active-healthy-ageing&pg=action_group_a3). This Action Group defined a number of deliverables: to manage functional decline and frailty through targeted intervention, to enhance participation and independence (public awareness materials, events and an interactive website), to promote systematic routine screening for frailty, to establish integrated pathways of care, to pursue research and methodology (to determine the effects of a healthy diet and to develop new nutritional supplements) and to manage demand.

### 3.2. The Importance of Micronutrient Intake in the First 1000 Days of Life

While micronutrient requirements vary with the different stages of life, micronutrient status has the greatest impact during the first 1000 days. This is because of the rapid growth, tissue and organ differentiation during this period of life, leading to very high nutrient needs per kg of bodyweight, while body stores are low and several gastrointestinal, metabolic, and renal functions related to nutrient turnover are still immature. The nutritional intake at this stage effects life-long health and has a major impact on short- and long-term outcomes [10]. The concept of metabolic programming was established almost four decades ago by Günther Dörner, who stated: ‘The concentrations of hormones, metabolites and neurotransmitters during critical early periods of development are capable of pre-programming brain development, functional disturbances and diseases as well as syndromes of reproduction and metabolism in human adulthood [11].

A recent systematic review and meta-analysis on poor vitamin D status in pregnancy has found that a low serum 25(OH)D level is independently associated with risk for gestational diabetes (pooled OR = 1.49), pre-eclampsia (1.79) and Small for Gestational Age (SGA) births: (1.85) [12]. In order to prevent birth defects, mandatory fortification of staple food with folic acid is currently applied in 58 countries. In “new Europe”, Kosovo has already started and Moldova is working on legislations to start fortification. Lately, a very large cohort study in Norway found maternal intake of folic acid before and during early pregnancy to be associated with a significantly reduced incidence of specialist-diagnosed autistic disorders (0.10% in children whose mothers took folic acid, 0.21% in those unexposed, adj. OR = 0.61) [13]. Micronutrients are essential for brain growth and MNDs cause a reduced cognitive outcome: stunted children have been shown to have a lower intelligence quotient (IQ) than non-stunted children [14]. Early iron deficiency alters the metabolism in the hippocampus and striatum, with lasting effects on monoamines (DA, 5–HT, NE), myelin proteins, and compaction. This can lead to long-term behavioral changes. A study into school progress in former iron-deficient children in Costa Rica at age 11–14 years shows that significantly more children who were previously iron-deficient are referred for special services or repeat a grade [15]. According to the European health claims for docosahexaenoic acid (DHA), maternal DHA intake contributes to the normal brain development and the development of the eye of the fetus and breastfed infants [16]. Further studies found pre- and postnatal fish and DHA intake to predict an improved IQ at school age [17]. Higher cord blood DHA was linked to less behavioral problems at 10 years [18] and its supply during pregnancy also modulated the infant’s immune response [19] and reduced infectious diseases in infants [20]. Breastfeeding, which provides preformed long-chain polyunsaturated fatty acid (LC-PUFA), is recommended as the optimal feeding choice for infants [21]. For infants that are not breastfed, a calculation of cost-effectiveness of standard and LC-PUFA enriched formula on QALYs and costs showed that LC-PUFA supplementation of infant formula represents an economically worthwhile prevention strategy, even if it is only based on the costs derived from hypertension-linked diseases in later life [22].

As late intervention may only have limited benefits, it is vital to optimize early nutrition for long-term health [23]. An adequate supply of critical nutrients (including Fe, Zn, I, folate, vitamin B_12_, vitamin A, vitamin D, and DHA) is essential before/throughout pregnancy and during lactation/infancy and early childhood.

### 3.3. Undernutrition in the Elderly

Average life expectancy has risen globally since 1950, currently reaching 57 years in Africa, 70 years in Latin America, 79 years in North America and 78 years in Europe. A geriatric population has several nutrition issues, from unintentional weight loss through poor appetite to malnutrition. It is reported that among those of 71 years or above, recommended intakes for fruits, vegetables, and whole grains are not met by around 70%, more than 80%, and more than 90%, respectively [24]. The prevalence of undernutrition changes from the age of 65 to 85 from 3.3% (in patients and healthy individuals), 7.8% (in patients in GP) and 33.8% (in home care patients) to 20.9%, 22.8%, and 32.8%, respectively [25]. As many as one in three older adults living independently are at risk of becoming undernourished [26]. A recent study found that 67% of patients in a European elderly care rehabilitation facility had a severe deficiency in 25(OH)D levels (below 10 ng/mL) [27].

The effects of undernutrition on the elderly are significant: increased hospital stays, increased risk of non-elective re-admission, higher incidence of complications (delirium, infections, pressure sores), impaired functional status and decreased muscle function [28]. In adult hospital patients, decreased hand-grip strength is a predictor of loss of functional (muscle) status [29]. Reduced muscle strength and fatigue can lead to falls, reduced ability to self-care, poor recovery from chest infection [30], and impaired mobility [31]. There are higher hospitalization rates, higher nursing home placement rates, a reduced quality of life and increased mortality. Disease-related malnutrition has been found to double the risk of mortality in hospital patients and to triple mortality in older patients in hospital and after discharge [32]. Older women with weight loss have increased rates of hipbone density loss, and the risk of subsequent hip fracture is doubled [33]. A Dutch study in undernourished elderly patients demonstrated that short-term nutritional intervention (energy- and protein-enriched diet, oral nutritional supplements, calcium-vitamin D supplement, telephone counseling by a dietitian), decreased the number of fall incidents [34]. The clinical criteria for frailty (unintentional weight loss/sarcopenia, weakness, poor endurance, and low activity) are associated with chronic undernutrition resulting in loss of weight and muscle mass and poor muscle function [35]. Without appropriate intervention, frail older people are likely to experience functional limitations and disability, increased morbidity and use of healthcare resources, and mortality. It was estimated that in the UK more than half of the expenditure on undernutrition goes to people aged ≥65 years of age, who account for only about 15% of the population [30]. Comparative calculations in other countries confirmed the higher costs in elderly. For example in The Netherlands, the total additional costs of disease-related malnutrition were about four times higher for patients of at least 60 years of age (€1.5 billion) than for patients in the age category of >18 and <60 years (€403 million) [36].

Malnutrition and/or weight loss in the elderly are cited as predictive variables for *Clostridium difficile* enterocolitis, postoperative pneumonia, intravascular device infection, catheter-associated urinary tract infection, and decubitus ulcer [37]. Yet, undernutrition in the elderly has no quantitative diagnosis, has no formal definition and is not recognized as a serious condition with major clinical and economic consequences. Comprehensive geriatric assessment including screening for malnutrition increases patients’ likelihood of surviving and returning to their own homes after an emergency admission to hospital [38]. Risk factor intervention strategies have demonstrated to result in significant reductions in the number and duration of episodes of delirium in hospitalized older patients [39]. More studies are needed, with multifaceted interventions, addressing the causes and risk factors as well as contributions of nutritional deficiencies [40]. To help combat this problem, it is recommended to adjust the nutrient density of the elderly diet according to the individual nutritional needs and intakes. Although ideally this would be achieved through a careful selection of a diverse daily nutrition intake, appropriate fortified foods and dietary supplements might prove to be a more promising approach. Given the magnitude of the problem, it is imperative to find ways [9] to close the gap between recommended and actual nutrients intakes in the elderly [41].

## 4. Established Evidence of Health and Economic Benefits of Selected Nutrients

**Figure 2:**
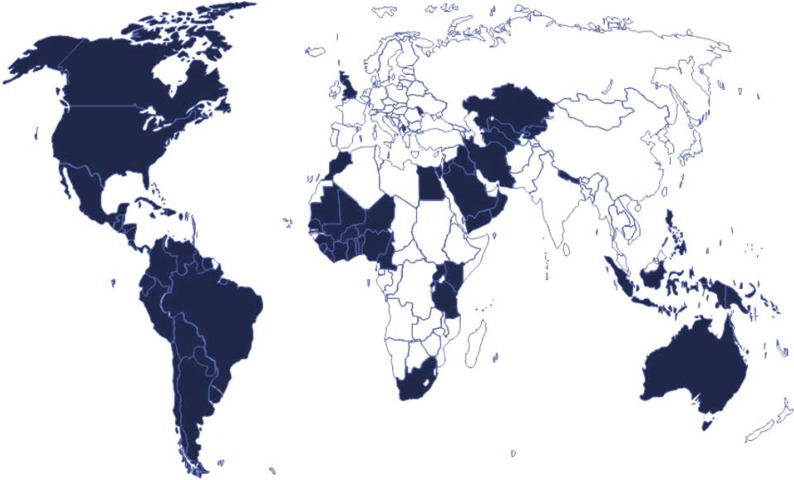
Grain Fortification Legislation—Countries in blue require fortification of wheat flour, maize flour, and/or rice (November 2013. Source: Flour Fortification Initiative).

The health and economic benefits of fortification or supplementation with certain micronutrients has been shown in many studies. Several countries have already implemented mandatory fortification programs with micronutrients. Health care costs can be reduced through fortification with e.g., folate. Folate plays a key role in DNA synthesis and delivering methyl groups. Both mechanisms are highly active during pregnancy and early embryogenesis, therefore folate deficiency during pregnancy is causally related to several developmental abnormalities. Neural tube defects (NTDs) are among the most common birth defects, with a huge and long-term social and economic burden. Since the closure of the neural tube takes place in the first few weeks after conception, an optimal folate status should be achieved before that time. Early supplementation with folic acid can prevent up to 70% of all NTDs [42], depending on baseline serum folate, the dose of folic acid supplemented and the time window available for prevention [43]. Folate has also been shown to prevent other birth defects or adverse birth outcomes such as congenital heart defects [44], orofacial clefts [45], low birth weight [46], and preterm births [47]. Women planning for pregnancy are recommended to take 0.4–0.8 mg folate/day starting at least 4 weeks before conception. Awareness and information campaigns that aimed at promoting folate usage among young women, increased the population awareness, but did not significantly affect practicing supplementation, neither were they effective in rising serum folate in the target group. Factors related to women’s age, education, and socioeconomic status limited the success of such campaigns in Canada [48]. The fortification with folate has been shown to improve folate status in the general population [49], to lower the incidence of NTDs, to be cost-effective [50], and safe for the population [51,52]. Low folate status is common in elderly people [53] and this was corrected in countries applying fortification (Figure 2) [54]. Higher folate status has been shown to protect against several age-associated diseases like coronary heart disease and cancer [53]. Mean dietary folate intake in Europe (180–250 μg/day) [55] does not provide sufficient protection against neural tube defects [56]. Effective national strategies, cooperation between the health system sectors, and legislations are needed in order to improve folate status of young women before conception.

Vitamin D insufficiency alone is potentially associated with a wide variety of diseases, *i.e.*, diseases of the musculo-skeletal system (associated with increased falls and fractures), immune system (associated with infections, allergies and tumors), endocrine system (associated with Type I and Type II diabetes), circulatory system (associated with hypertension, stroke, heart attack, and heart failure), and diseases of the nervous system (associated with multiple sclerosis, depression, and Alzheimer’s disease) [57]. The European Society for Clinical and Economic Aspects of Osteoporosis and Osteoarthritis (ESCEO) convened a meeting to provide recommendations for clinical practice, to ensure the optimal management of elderly and postmenopausal women with regard to vitamin D supplementation. Finding that patients with reduced vitamin D levels have increased bone turnover, bone loss, and possible mineralization defects, the ESCEO recommended supplementation of 800 to 1000 IU/day of vitamin D when the serum level is below 50 nmol/L. The same society has also advised that this threshold should be higher (75 nmol/L) for fragile, elderly subjects who are at increased risk of falls and fracture [58]. Furthermore, data from a recent meta-analysis suggest a nonlinear decrease in mortality risk as circulating 25(OH)D increases, with optimal concentrations of approximately 75–87.5 nmol/L [59]. A recent study shows that, especially for France and Sweden, the societal burden of hip fractures associated with low calcium intake is quite substantial. This study calculated that the yearly number of DALYs lost was 6263 for France, 1246 for Sweden, and 374 for The Netherlands. The corresponding total costs that might potentially be avoided are about 129 million, 34 million, and 6 million Euros, in these countries, respectively [60]. More models for assessing and calculating costs related to inadequate nutrition as well as the assessment of the efficiency of nutritional programs are necessary.

## 5. Pertinent Considerations in Providing Nutritional Solutions

### 5.1. Disease-Related Malnutrition and the Economic Value of Medical Nutrition

Disease-related malnutrition affects 33 million people in Europe and costs the European healthcare system €170 billion per year [61]. According to Eurostat (2010), there are 500 million people in Europe, with a GDP of €12.279 trillion, the total expenditure on health is approximately 10.4% of the GDP, or €1.28 trillion. In the management of malnutrition, early identification is key and screening should be routine practice [4]. Food for Special Medical Purposes (FSMP), also known as medical nutrition (or clinical nutrition), is a range of innovative nutritional products to be used under medical supervision and underpinned by scientific research. The nutrition administered is of unique compositions of specific nutrients, in formats and compositions to meet specific needs and administered enterally, which includes oral nutritional supplements as well as enteral tube feeding. In Europe, medical nutrition products are regulated by the Commission Directive 1999/21/EC on dietary foods for special medical purposes. FSMP has demonstrated to have nutritional, clinical, and functional as well as economic benefits. For example, in the Netherlands the total additional costs of managing adult patients with disease-related malnutrition were estimated to be €1.9 billion in 2011, which equals 2.1% of the total national health expenditure [36]. A health economic analysis assessing the cost-effectiveness of oral nutritional supplements in undernourished Dutch abdominal surgery patients showed that using oral nutritional supplements resulted in a €252 (7.6%) cost saving per patient, leading to an annual costs saving of at least €40.4 million per year. These costs savings were due to a reduction in hospitalization costs from €3318 to €3044 per patient and corresponds with 0.72 days reduction in length of stay. [62]. Another medical nutrition economic study on oral nutritional supplements in the Netherlands demonstrated a cost saving of €12,986 million (4.7% savings) in community dwelling for elderly patients suffering from disease-related malnutrition. The additional costs of oral nutritional supplements (€57 million) were more than balanced by the reduction in the total costs of disease-related malnutrition due to a reduction of re-/hospitalization (€70 million) [63]. Similar financial benefits of oral nutritional supplements in hospital have been demonstrated in Denmark and other European countries [64]. According to a recently published eleven-year retrospective (2000 to 2010) study in the US, the use of oral nutritional supplements decreases length of stay, episode cost, and 30-day readmission risk in the inpatient population [65]. These findings were confirmed by two recent systematic reviews stating that the use of FSMP in the management of disease-related malnutrition, for inpatients as well as outpatients, is efficient from an economic perspective [66,67].

### 5.2. Fortification of Affordable Products for Emerging Economies

Micronutrient deficiency as well as undernutrition as a whole is a common public health problem in developing countries, especially for infants, young children, and women of reproductive age. In a recent systematic review and meta-analysis, the effects of micronutrient fortified milk and cereal food on the health of the younger age groups have been evaluated, using data from randomized controlled trials. In the Philippines, 36% of all children between the ages of 0.5–5 years, suffer from malnutrition and micronutrient deficiencies, resulting in costs from disease of approximately 1 billion USD. Compared to non-fortified food, iron plus multi-micronutrient fortification increases hemoglobin levels by 0.87 g/dL and reduces risk of anemia by 57%. It was also found that iron plus multi-micronutrient-fortification is more effective than iron only fortification for hematologic outcomes like hemoglobin levels. The study demonstrates that multi-nutrient fortified milk and cereal products are an effective option to reduce anemia in children up to 3 years of age in developing countries, resulting in a reduction of common morbidities in pre-school children. Reducing anemia by 50% would mean a GDP gain of 0.2% (ca. 0.35 billion USD) or 4.5% of health costs in the Philippines [68]. This example clearly shows the benefits of improved early nutrition for the individuals and for the economies of their countries.

## 6. Conclusions and Pending Issues to Reduce Health Care Costs

Nutrition is very complex, as it comprises a mixture of nutrients acting in concert and dependent on one another. Nevertheless, a significant scientific and medical consensus exists about the importance of an appropriate level of macro- and micronutrient intake throughout the course of life to support growth, foster health, and prevent the onset of disease. Micronutrient requirements vary with the different stages of life with adequate intakes having the highest impact during the first 1000 days of life. Later in life, micronutrient intake has to be adjusted to the specific requirements of the elderly and those living in particular settings such as hospitals and institutions. Other demographic groups are also at risk of nutrient deficiencies and inadequate intake, like pregnant and breastfeeding women, certain patient groups, and low-resource communities. Moreover, recent changes in life-style and eating patterns, along with increasing dependency on pre-cooked and processed foods, require more attention to nutrition as a key factor in determining human health. Appropriate micronutrient intake—as part of a balanced daily diet and in combination with a healthy lifestyle—supports health and well-being. The presence of undernutrition and micronutrient deficiencies in the developing world is well established, but a growing body of evidence indicates that micronutrient deficiencies also exist in the developed world, as does malnutrition, negatively affecting health, wellbeing, and economic status of significant sections of society. These nutritional deficiencies are often ignored and underappreciated despite being a global issue of rising importance with devastating consequences for individuals, communities and national economies. It is critical to continue developing nutritional solutions and economic models that address the value of nutrition interventions in improving public health as well as the impact thereof on health care budgets—evidence is very encouraging, however key stakeholders need to engage to make it happen.

**Figure 3:**
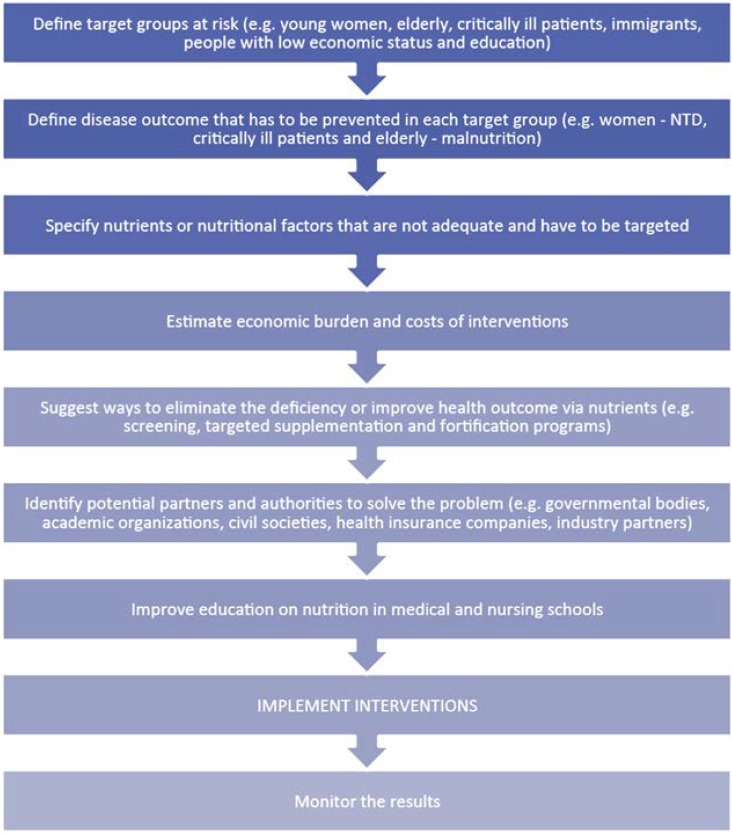
Suggested strategic steps to reduce the burden of hidden hunger in developed countries

The extensive scientific knowledge currently available needs to be translated into cost-effective, practical public health solutions. These may include fortification and/or supplementation and specific delivery mechanisms should be assessed on a case-by-case basis. The health and economic benefits of fortification or supplementation with certain micronutrients—e.g., vitamin A (to reduce infant mortality), iodine (to reduce goiter), vitamin D (primarily against rickets), and folic acid (primarily to reduce neural tube defects)—as of medical nutrition are well-established. Many countries already have mandatory fortification programs in place regarding micronutrients. The absence of such programs in Europe (for folic acid and vitamin D) should be scrutinized in the light of the overwhelming evidence of the proven benefits of these fortification/supplementation programs. While the health and economic benefits of fortification or supplementation with vitamin D and folic acid are clear, more research is required in the context of other micronutrients. Micronutrient fortification and supplementation programs in the developing world should be scaled up to national level with the necessary support from donors. It is essential to give undernutrition the same high-level attention as is given to overnutrition and to translate existing evidence into public health actions. Malnutrition, meaning undernutrition in this article, is a major public health concern, mostly not visible in our increasingly overweight society but has serious health and economic consequences that cannot be ignored. The costs due to malnutrition in Europe exceed those related to obesity [69]: this will require public-private partnerships involving governments, academia, civil society and the private sector—four groups that have a key role in providing the necessary nutritional solutions (Figure 3).
